# Efficient siRNA delivery and gene silencing using a lipopolypeptide hybrid vector mediated by a caveolae-mediated and temperature-dependent endocytic pathway

**DOI:** 10.1186/s12951-019-0444-8

**Published:** 2019-01-22

**Authors:** Hironori Kasai, Kenji Inoue, Kentaro Imamura, Carlo Yuvienco, Jin K. Montclare, Seiichi Yamano

**Affiliations:** 1 Department of Prosthodontics, New York University College of Dentistry, New York, NY 10010 USA; 2 Department of Periodontology, Tokyo Dental College, Tokyo, Japan; 3 Department of Chemical and Biomolecular Engineering, New York University Tandon School of Engineering, Brooklyn, NY 11201 USA; 4 Department of Chemistry, New York University, New York, NY 10003 USA; 5 Department of Biomaterials, New York University College of Dentistry, New York, NY 10010 USA; 6 Department of Radiology, New York University School of Medicine, New York, NY 10010 USA

**Keywords:** Gene delivery, Non-viral vector, Small interfering RNA, Transfection, RNA interference

## Abstract

**Background:**

We developed a non-viral vector, a combination of HIV-1 Tat peptide modified with histidine and cysteine (mTat) and polyethylenimine, jetPEI (PEI), displaying the high efficiency of plasmid DNA transfection with little toxicity. Since the highest efficiency of INTERFERin (INT), a cationic amphiphilic lipid-based reagent, for small interfering RNA (siRNA) transfection among six commercial reagents was shown, we hypothesized that combining mTat/PEI with INT would improve transfection efficiency of siRNA delivery. To elucidate the efficacy of the hybrid vector for siRNA silencing, *β*-*actin* expression was measured after siRNA *β*-*actin* was transfected with mTat/PEI/INT or other vectors in HSC-3 human oral squamous carcinoma cells.

**Results:**

mTat/PEI/INT/siRNA produced significant improvement in transfection efficiency with little cytotoxicity compared to other vectors and achieved ≈ 100% knockdown of *β*-*actin* expression compared to non-treated cells. The electric charge of mTat/PEI/INT/siRNA was significantly higher than INT/siRNA. The particle size of mTat/PEI/INT/siRNA was significantly smaller than INT/siRNA. Filipin III and β-cyclodextrin, an inhibitor of caveolae-mediated endocytosis, significantly inhibited mTat/PEI/INT/siRNA transfection, while chlorpromazine, an inhibitor of clathrin-mediated endocytosis, did not inhibit mTat/PEI/INT/siRNA transfection. Furthermore, the transfection efficiency of mTat/PEI/INT at 4 °C was significantly lower than 37 °C.

**Conclusions:**

These findings demonstrated the feasibility of using mTat/PEI/INT as a potentially attractive non-viral vector for siRNA delivery.

## Background

RNA interference (RNAi), such as small interfering RNA (siRNA)-mediated gene silencing has great potential to generate entirely new therapeutic paradigms in many diseases. The mechanistic basis of RNAi is that 19- to 23-nucleotide long double-stranded RNA fragments with two-nucleotide 3′overhanging ends are processed by the RNA-induced silencing complex (RISC) to yield single-stranded RNA molecules. The RISC bound with single-stranded RNA molecules then binds and degrades the complementary mRNA. Since it was first reported that siRNAs are mediators of gene-specific silencing, viral and non-viral delivery systems have been developed for multiple purposes such as gene regulation, gene therapy or biotechnologies. Viral vectors are efficient but some risks may exist for the host such as the possibility of uncontrolled cell proliferation of transduced cells, immune reactions to viral particles, and inflammation of the transduced tissue. Therefore, non-viral vectors represent an alternative because of their reduced safety concerns and the relatively more convenient preparation techniques [1, 2].

Cell-penetrating peptides (CPP) are a class of non-viral delivery vectors that has been used for the intracellular delivery of various bioactive cargos. The Tat protein of human immunodeficiency virus type-1 (HIV-1), the first finding CPP, is the most frequently used cell-permeable peptide. A number of Tat peptides have been successfully used to deliver drugs, protein, DNA and siRNA into cell [3–5]. Notably, several papers reviewing the delivery of siRNA using Tat peptide have recently been reported [6–10]. Lo and Wang [11] demonstrated significant improvement in gene transfection efficiency using a modified Tat peptide covalently fused with ten histidine and two cysteine residues (mTat) when compared to unmodified Tat. Gene transfection was improved because interpeptide disulfide bonds, formed by air oxidation upon binding to gene, led to enhanced stability of peptide/gene complexes.

The cationic polymer, polyethylenimine (PEI) is a useful delivery vehicle for oligonucleotides and ensures effective oligonucleotides delivery with low toxicity in spite of relatively short duration of gene expression. Because PEI forms stable complexes with oligonucleotides, the positively charged particles are able to interact with anionic proteoglycans at the cell surface and enter cells by endocytosis [12]. A previous study by our group showed that mTat with commercial PEI, jetPEI results in much more efficient gene transfer in medium supplemented with serum compared to several commercial reagents in vitro [13].

It has recently been reported that INTERFERin (INT), a non-liposomal cationic amphiphilic lipid-based transfection reagent, is one of the most efficient reagents for siRNA delivery [14, 15]. We found that INT results in very efficient gene transfer compared to several commercial reagents in vitro, similarly to these previous studies. In the present study, we therefore focused on the potential of INT and proposed a hypothesis that combining mTat/PEI with INT would improve siRNA transfection efficiency. To investigate our hypothesis, we conducted transfections in HSC-3 human oral squamous cell carcinoma cells using preparations of mTat/PEI with INT and siRNA targeting *β*-*actin* compared to several commercial reagents. We also examined the cytotoxicity of the hybrid vectors toward HaCaT human keratinocytes. In addition, we investigated the mechanism of intracellular delivery of mTat/PEI/INT/siRNA. Particularly, we examined the surface charge and size of the complexes and whether the mode of delivery was via clathrin- or caveolae-mediated endocytosis. We also explored the effects of temperature on transfection. Furthermore, we observed the morphological characteristics, cellular uptake, and localization of the complexes.

## Results

### Optimization of each vector to siRNA, and mTat, PEI and siRNA to INT ratio

**Fig. 1:**
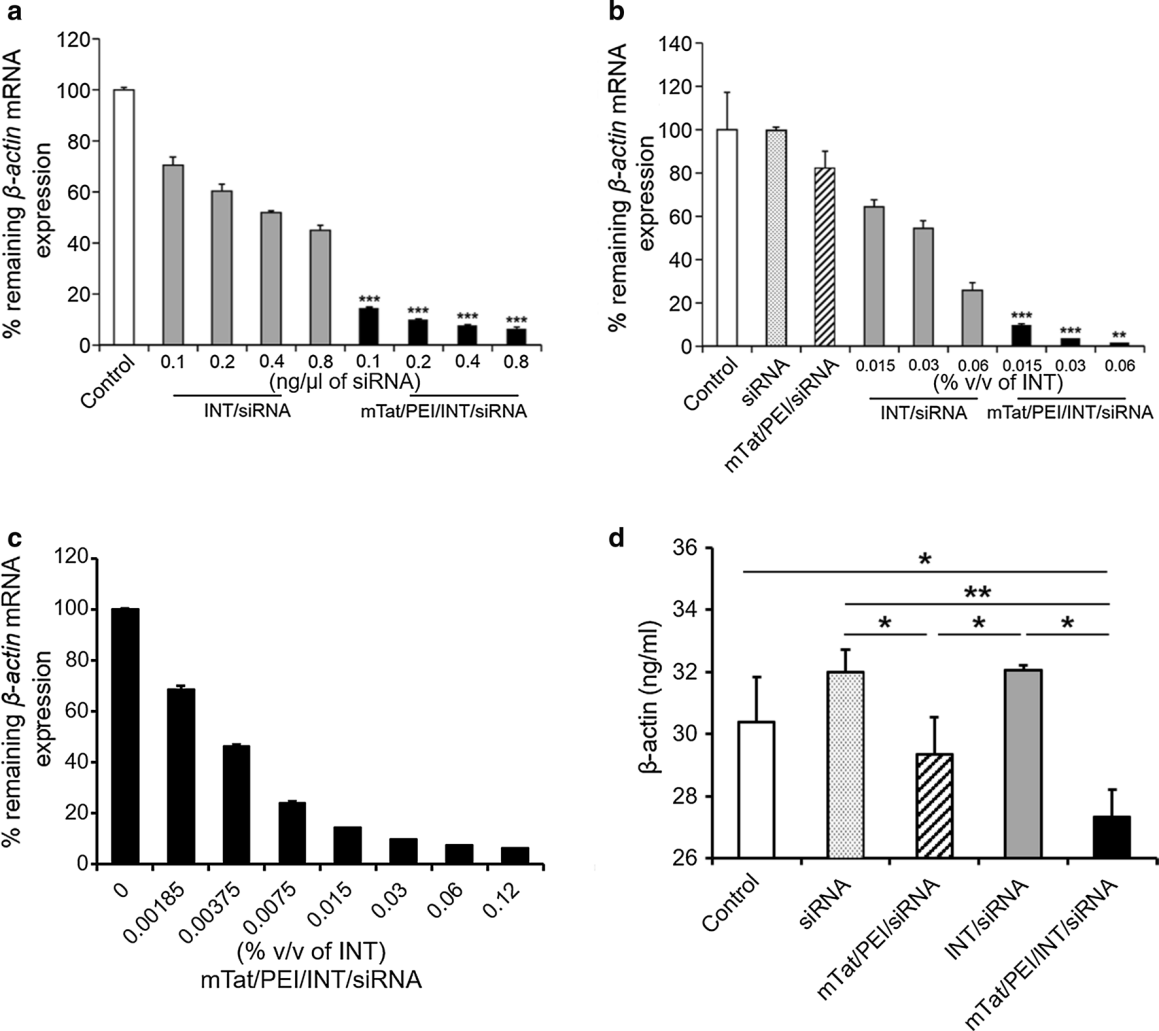
In vitro gene silencing efficiency of various concentrations of siRNA (0.1, 0.2, 0.4 and 0.8 ng/µl) with/without mTat/PEI, INT, and mTat/PEI/INT in HSC-3 cells. *In vitro* transfection efficiency of siRNA with/without mTat/PEI, INT (0.015, 0.03 and 0.06% v/v), and mTat/PEI/INT (0.015, 0.03, and 0.06% v/v) in HSC-3 cells (**a**). *β*-*actin* mRNA was measured by QRT-PCR and then % remaining *β*-*actin* mRNA expression was calculated based on control as 100% (**b**). Transfection efficiency of siRNA with mTat/PEI/INT at the different concentration. siRNA concentration was 0.8 ng/µl (**c**). β-actin protein was measured in the cell lysate of HSC-3 cells transfected with siRNA with/without mTat/PEI, INT, and mTat/PEI/INT incubated for 48 h (**d**). siRNA concentration was 0.8 ng/µl and INT concentration was 0.06% v/v. Significant difference between INT/siRNA and mTat/PEI/INT/siRNA, *p < 0.05, **p < 0.01 and ***p < 0.001

To investigate the gene silencing efficiency of our combined vector/siRNA complexes, HSC-3 cells were transfected with INT/siRNA and mTat/PEI/INT/siRNA. The transfection efficiency of vector-siRNA complexes was analyzed relative to *β*-*actin* mRNA expression by QRT-PCR. Before evaluating transfection efficiencies of the mTat, PEI and INT vector formulations in cell lines, optimal transfection conditions were identified by varying the amounts of siRNA encoding *β*-*actin* in HSC-3 cells. In this optimization experiment, optimal transfection efficiency was achieved with mTat/PEI/siRNA ratio of 5:1:1 (w/w). Compared to non-treated cells, both INT/siRNA and mTat/PEI/INT/siRNA inhibited *β*-*actin* mRNA expression in HSC-3 cells in a dose-dependent manner with increasing siRNA concentration (Fig. 1a). Furthermore, mTat/PEI/INT/siRNA significantly suppressed the target gene expression compared to INT/siRNA. The mTat/PEI/INT/siRNA complex also inhibited the mRNA expression in a dose-dependent manner by increasing INT concentration (Fig. 1b, c). In particular, HSC-3 cells transfected with mTat/PEI/INT/siRNA achieved almost 100% knockdown of *β*-*actin* mRNA expression at the highest concentration of INT used. These ratios were used for all subsequent studies. To further confirm the expression levels of β-actin protein in HSC-3 were measured by ELISA. The mTat/PEI/INT/siRNA complex shows significantly inhibit β-actin protein in HSC-3 compare with other groups (Fig. 1d).

### Cell viability of siRNA targeting β-actin with/without INT and mTat/PEI/INT in HaCaT cells

**Fig. 2:**
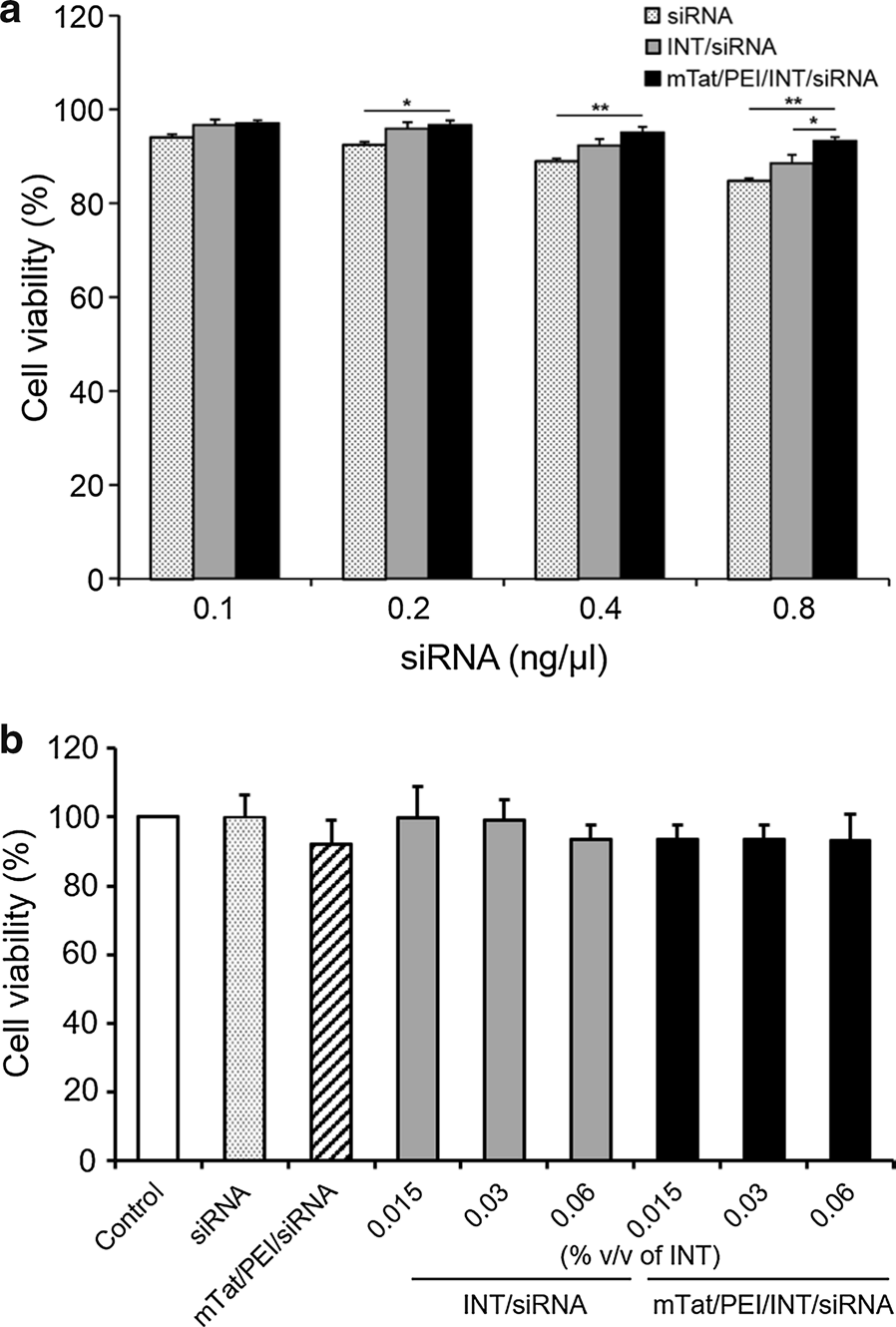
Cell viability of siRNA (0.1, 0.2, 0.4, and 0.8 ng/μl) with/without INT and mTat/PEI/INT in HaCaT cells evaluated by MTT assay compared to control as 100% (**a**). Cell viability of INT (0.015, 0.03 and 0.06% v/v) and siRNA (0.8 ng/ml) with/without mTat/PEI/INT in HaCaT cells was evaluated by MTT assay compare to control as 100% (**b**). *p < 0.05 and **p < 0.01

For the determination of cytotoxicity, composition and preparation of complexes and transfection conditions were the same as those used in the transfection efficiency studies. Compared to siRNA alone and INT/siRNA, the highest degree of cell viability was found after transfection with mTat/PEI/INT/siRNA in HaCaT cells (Fig. 2a). Significant differences were found between groups for siRNA alone, INT/siRNA and mTat/PEI/INT/siRNA. Besides, it is interesting that while the cytotoxicity was not so high, siRNA alone was most cytotoxic to HaCaT cells compared to the other complexes. Furthermore, we evaluate the cytotoxicity at the different concentration of INT under the same siRNA concentration. There was no significant difference between all groups. The mTat/PEI/INT/siRNA complex (0.015, 0.03 and 0.06% v/v) shows above 90% of cell viability (Fig. 2b).

### Comparative efficiency and cell viability of mTat/PEI/INT versus several commercial reagents

**Fig. 3:**
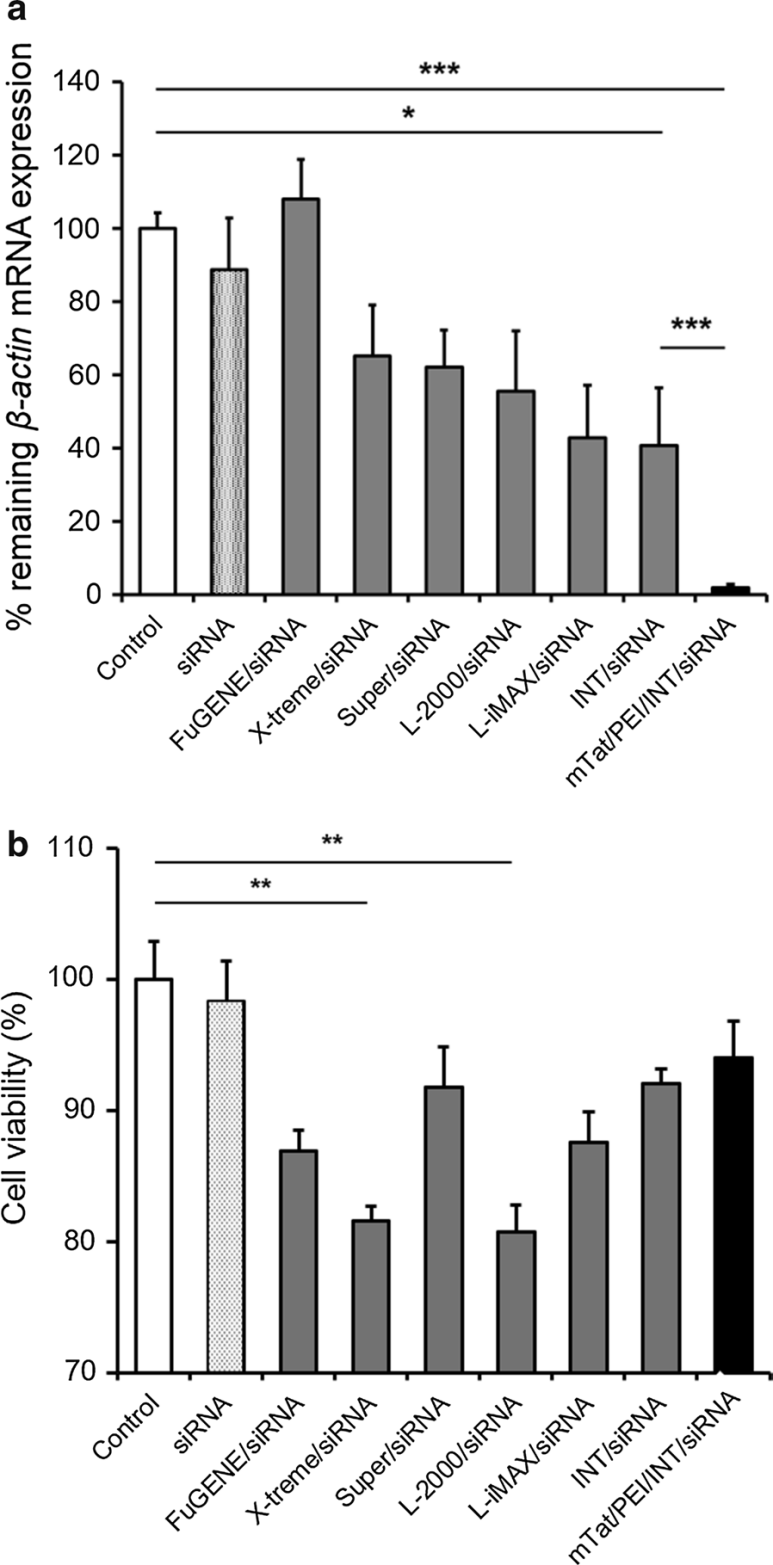
Comparative transfection efficiency of FuGENE HD (FuGENE), X-tremeGENE (X-treme), SuperFect (Super), Lipofectamine 2000 (L-2000), Lipofectamine RNAiMAX (L-iMAX), INT, and mTat/PEI/INT using siRNA targeting *β*-*actin* in HSC-3 cells. *β*-*actin* mRNA was measured by QRT-PCR and then % remaining *β*-*actin* mRNA expression was calculated based on control as 100% (**a**). Cell viability of each of the reagent groups in HaCaT cells were evaluated by MTT assay under the same siRNA and transfection reagent volume (**b**). *p < 0.05, **p < 0.01, and ***p < 0.001

To compare the transfection efficiency among several commercial non-viral vectors in vitro, we measured *β*-*actin* mRNA expression in HSC-3 cells at 48 h after transfection by QRT-PCR. As the result, INT markedly inhibited *β*-*actin* mRNA expression compared to control, siRNA alone, FuGENE HD, X-tremeGENE, SuperFect Lipofectamine 2000, or Lipofectamine RNAiMAX. The result showed that the combination of mTat/PEI/INT/siRNA was significantly downregulated expression of *β*-*actin* compared to any other several commercial reagents (*p* < 0.001) (Fig. 3a). In addition, the largest amount of viable cells was found after transfection with mTat/PEI/INT/siRNA complex in HaCaT cells (Fig. 3b). In contrast, X-tremeGENE and Lipofectamine 2000 were significantly lower than control (*p* < 0.01).

### The effect of mTat/PEI/INT/siRNA on particle charge and size

**Fig. 4:**
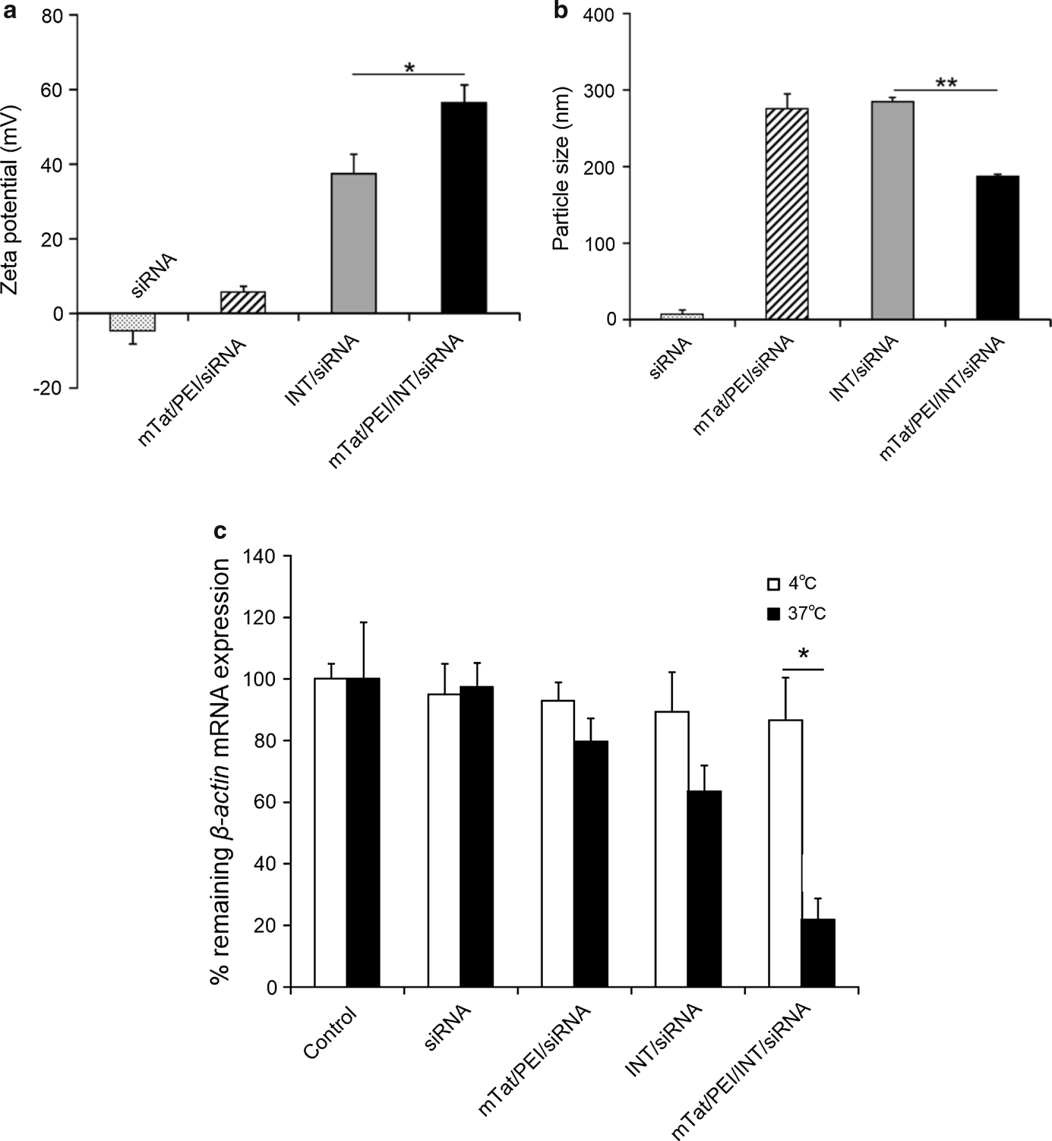
Zeta potentials (**a**) and particle sizes (diameter) (**b**) of siRNA, siRNA with Tat/PEI, INT, and siRNA with mTat/PEI/INT. Effects of temperature on siRNA transfection efficiency with mTat/PEI, INT, or mTat/PEI/INT in HSC-3 cells at 4 °C (white bars) or 37 °C (black bars) (**c**). *p < 0.05 and **p < 0.01

The zeta potentials of siRNA and each vector complex were determined. The zeta potential of the siRNA alone was negative with a value of − 4.7 ± 3.7 mV (Fig. 4a). A change in the zeta potential from negative to positive was observed when siRNA was combined with mTat/PEI, INT, or mTat/PEI/INT. The most positive value of the zeta potential was mTat/PEI/INT/siRNA, followed by INT/siRNA and mTat/PEI/siRNA (56.3 ± 4.7, 37.4 ± 5.1, and 5.6 ± 1.5 mV, respectively; Fig. 4a). The zeta potential of mTat/PEI/INT/siRNA was significantly higher than that of INT/siRNA (*p* < 0.05). In addition, the effect of mTat/PEI mixed with INT on particle size was determined. The z-average diameter of mTat/PEI/INT/siRNA (187 ± 3 nm) was significantly smaller than that of INT/siRNA (286 ± 5 nm) (*p* < 0.01) (Fig. 4b).

### Effects of temperature on the internalization of mTat/PEI/INT/siRNA

To determine whether the mTat/PEI/INT-mediated transfection under investigation in this study followed an energy-dependent or -independent pathway, we examined transfection efficiency at different temperatures (4 °C and 37 °C) using HSC-3 cells. Figure 4c shows that the *β*-*actin* mRNA expression level with mTat/PEI/INT/siRNA was about 65% recovered when transfection was carried out at 4 °C rather than at 37 °C (*p* < 0.05). The results indicate that low temperature directly inhibits transfection efficiency, suggesting that mTat/PEI/INT enters HSC-3 cells in an energy-dependent pathway. We found no significant difference in the mRNA expression between 4 and 37 °C in the other groups.

### The effect of caveolae- and clathrin-mediated endocytosis inhibitors on transfection of mTat/PEI/INT/siRNA in HSC-3 cells

**Fig. 5:**
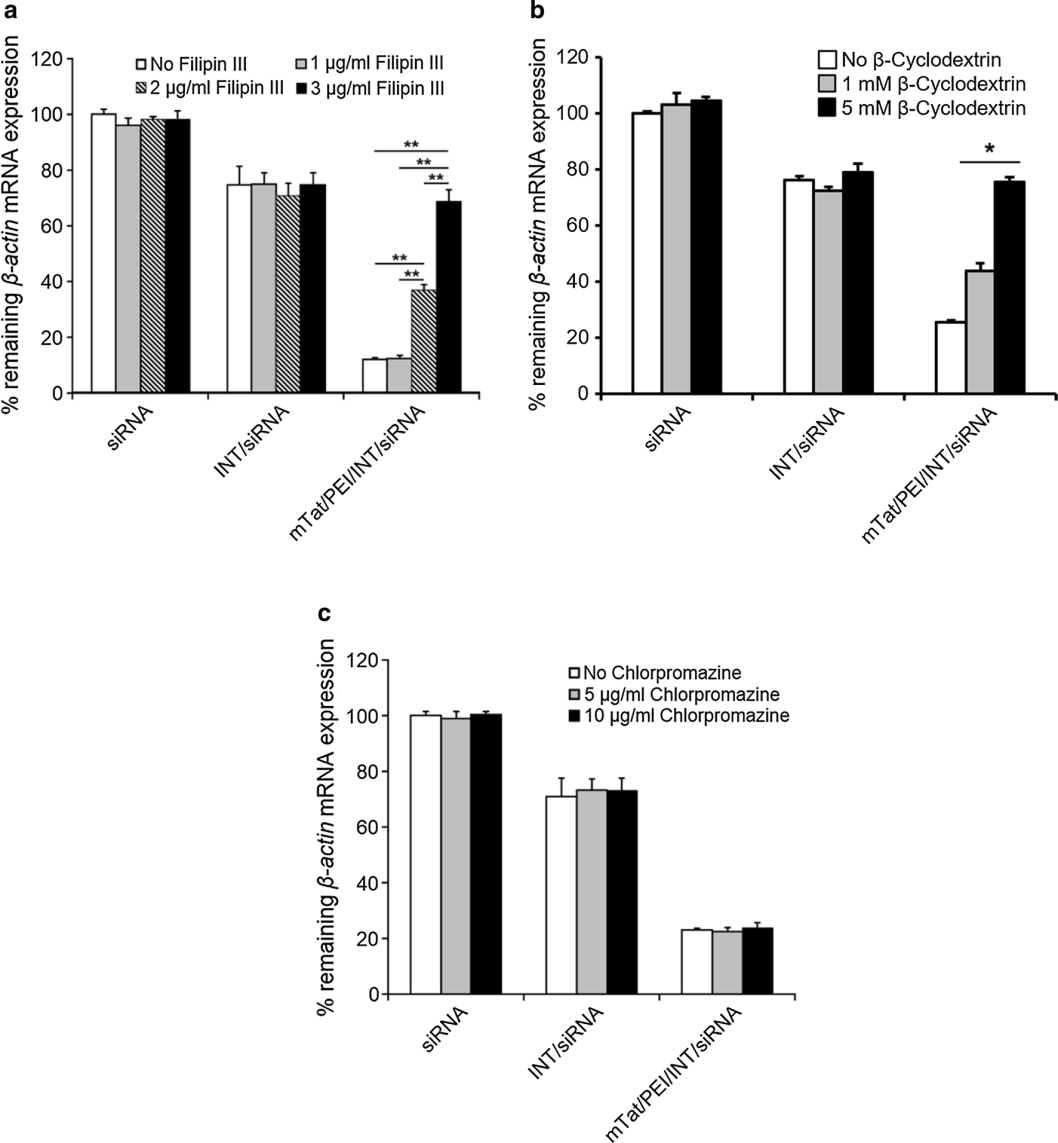
Effects of several concentrations of inhibitors, filipin III (**a**), β-cyclodextrin (**b**), and chlorpromazine (**c**), on internalization of siRNA with/without INT and mTat/PEI/INT in HSC-3 cells. *β*-*actin* mRNA was measured by QRT-PCR and then % remaining *β*-*actin* mRNA expression was calculated based on control as 100%. *p < 0.05 and **p < 0.01

We investigated the effects of inhibitors of caveolae-mediated endocytosis (filipin III and β-cyclodextrin) and of clathrin-mediated endocytosis (chlorpromazine) on the transfection efficiencies of mTat/PEI/INT/siRNA complexes by HSC-3 cells. Transfection efficiency with INT or mTat/PEI/INT was measured by QRT-PCR in the presence and absence of filipin III, β-cyclodextrin, or chlorpromazine (Fig. 5). No significant change of knockdown of mRNA expression was observed when siRNA alone and INT/siRNA complex were treated with filipin III compared to control. In contrast, the mRNA knockdown of mTat/PEI/INT/siRNA treated with filipin III (2 or 3 µg/ml) was significantly diminished in a dose-dependent manner with increasing filipin III concentration (*p* < 0.01) (Fig. 5a). Furthermore, the mRNA knockdown of mTat/PEI/INT/siRNA treated with β-cyclodextrin (5 mM) was significantly diminished (*p* < 0.05) (Fig. 5b). On the other hand, treatment of the cells with chlorpromazine did not affect the knockdown of mRNA expression (Fig. 5c). These results suggest that mTat/PEI/INT-mediated transfection may be associated with caveolae-mediated endocytosis pathway.

### The morphological analysis of complex nanostructures with β-actin siRNA

**Fig. 6:**
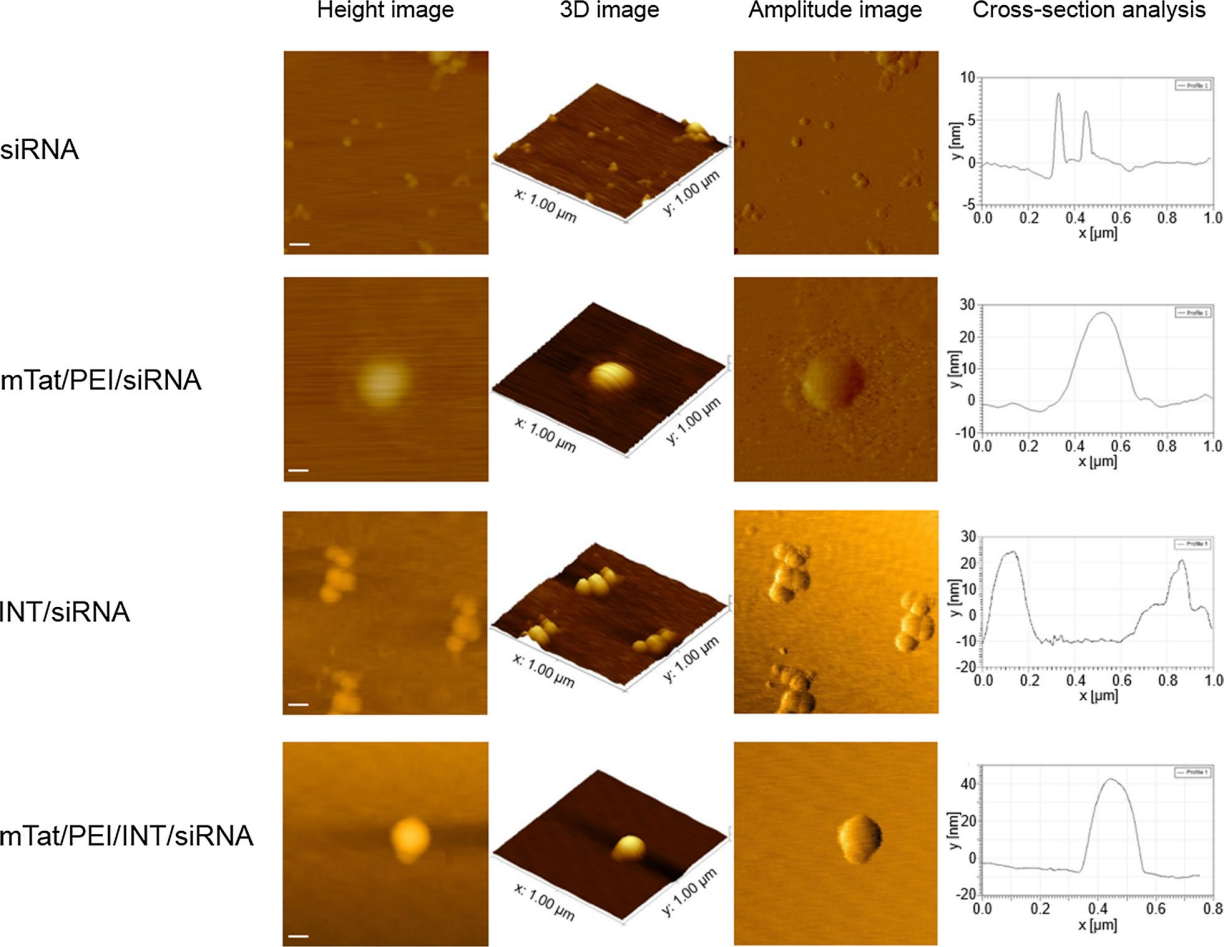
Detailed structure and morphology of free siRNA, mTat/PEI/siRNA, INT/siRNA and mTat/PEI/INT/siRNA by AFM. AFM images: height image, 3D image, amplitude image, and cross-section analysis (left to right) of the complexes. The frames of height images and amplitude images are 1 × 1 µm^2^. Scale bars are 100 nm

The structure of complex nanostructures with siRNA targeting *β*-*actin* was further elucidated using AFM. Figure 6 represents AFM images of the β-actin siRNA with/without vectors. The mTat/PEI/siRNA imaging showed the particle surrounded by a bilayer (Fig. 6, second row). Interestingly, different from the smooth edge of particles formed by mTat/PEI/INT/siRNA (Fig. 6, last row), we could observe a granular morphology of INT/siRNA complexes (Fig. 6, third row). The mTat/PEI/INT/siRNA complex appeared as almost spherical particles (Fig. 6, last row). The particle size for the mTat/PEI/INT/siRNA complex visualized by AFM was also in accord with the result of DLS.

### Cellular uptake and location of labeled siRNA

**Fig. 7:**
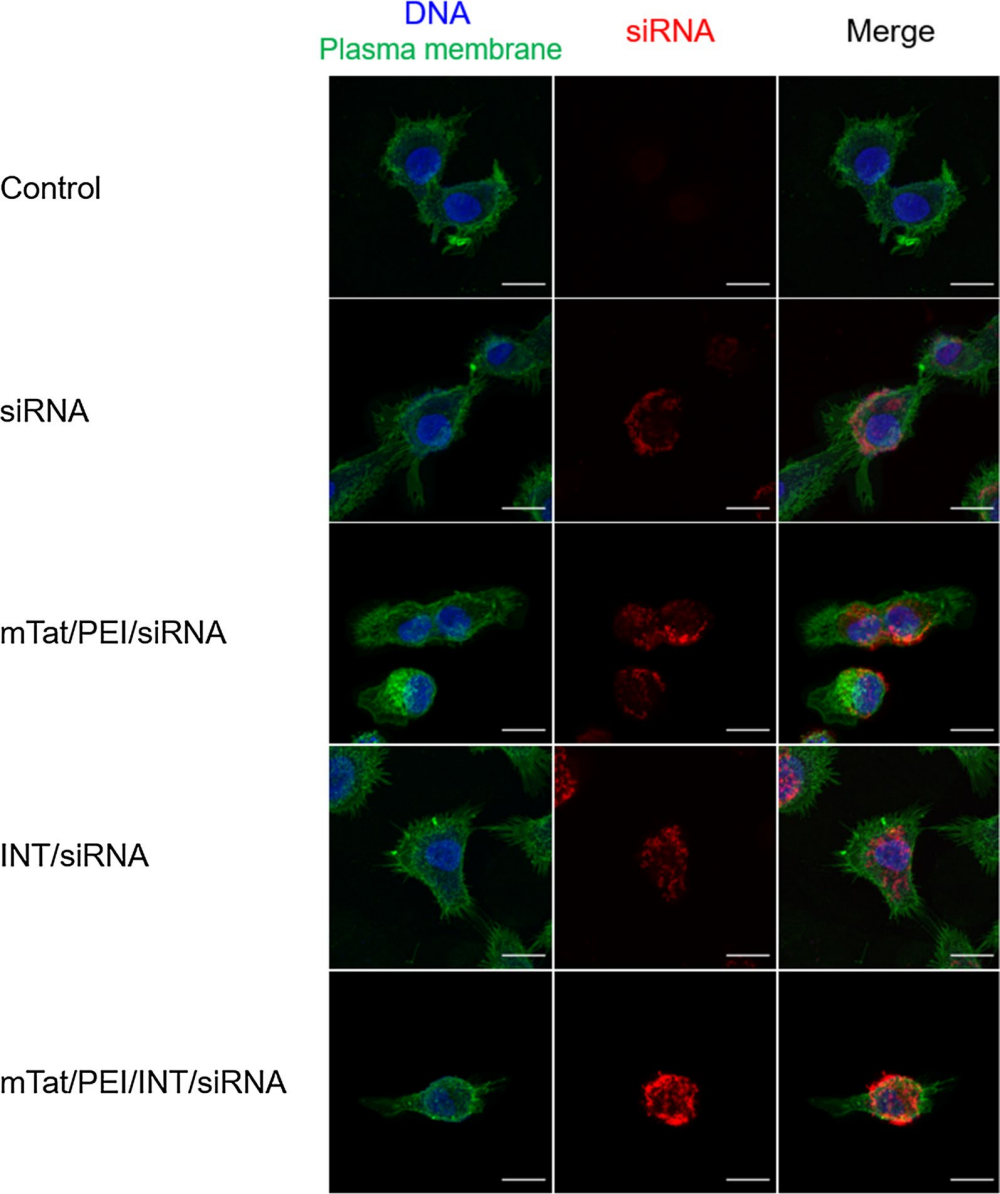
Confocal fluorescence microscopy images of HSC-3 cells treated with/without siRNA, mTat/PEI/siRNA, INT/siRNA, or mTat/PEI/INT/siRNA, respectively. The cell nuclei were stained with DAPI (blue), and the plasma membranes were stained with CF™ 488A (Alexa Fluor 488) Wheat Germ Agglutinin (green). The Cy5 labeled siRNAs appear in red. Scale bars are 10 µm

Cellular uptake efficiencies of siRNA alone, mTat/PEI/siRNA, INT/siRNA or mTat/PEI/INT/siRNA was evaluated to visualize the internalization of Cy5 labeled siRNA and their following intracellular distributions using confocal fluorescence microscopy (Fig. 7). As shown in Fig. 7 the second row, siRNA was seen on the cell membrane stingingly. The imaging of mTat/PEI/siRNA showed that the labeled siRNA surrounded the cell membrane and was untaken into cytosol slightly (Fig. 7, third row). In contrast, the INT/siRNA complex was distribution of the siRNA exclusively in the cytoplasm. However, the surface localization of siRNA was fewer than the imaging of mTat/PEI/siRNA (Fig. 7, fourth row). Importantly, the imaging of mTat/PEI/INT/siRNA showed that the siRNA complexes more strongly localized not only in the cytoplasm but also on the cell membranes compared to others (Fig. 7, last row).

## Discussion

A large variety of transfection reagents have been used to deliver DNA and siRNA into cells to achieve genetic manipulations, and may eventually enable non-viral gene therapy. However, it is difficult to optimize the reagents and to achieve favorable results due to its cytotoxicity and stability. Several recent studies have suggested that lipid-based reagent INT is one of the most efficient reagents for siRNA delivery [14, 15]. In the present study, we showed that INT resulted in the highest silencing effect of siRNA among six commercial reagents. Furthermore, we demonstrated that a combination of HIV-1 mTat with the cationic polymer PEI, INT and siRNA significantly enhanced transfection and gene silencing compared to INT alone or mTat/PEI in HSC-3 cells. In particular, mTat/PEI with 0.06% (v/v) INT and 0.8 ng/μl β-actin siRNA accomplished almost 100% knockdown of *β*-*actin* mRNA expression compared to non-treated cells. This study shows for the first time that a novel lipopolypeptide hybrid vector dramatically enhances gene silencing efficiency in vitro. Although it is not completely understood how mTat/PEI/INT/siRNA complexes yield higher transfection efficiency and gene silencing, the sequence of mTat peptide may function similar to the condensing agent, protamine sulfate, which can significantly increase transfection efficiency of cationic polymer-gene complexes and cationic lipid-gene complexes [16, 17]. In our previous study, we indicated that a combination of mTat with PEI and plasmid DNA significantly enhanced transfection compared to mTat alone or PEI alone in some different cell types [13]. We have also previously demonstrated that mTat combined with the cationic lipids results in very efficient gene transfer across a range of cell lines [18]. It is thought that inter-peptide disulfide bonds formed by air oxidation upon binding to gene can lead to enhanced stability of peptide/gene complexes. More recently, many research groups have reported that the inclusion of hydroxyl, carboxyl, and amino groups in polycations can help to reduce cytotoxicity and improve gene delivery efficiency [19–21]. Additionally, some reports have suggested that modification by lipids to PEI is beneficial for efficient gene delivery [14, 22]. It has lately been considered that polymer-lipid hybrid nanoparticles possess both the characteristics of liposomes and nanoparticles, such as high stability, biocompatibility and controlled release properties, which mean this type of nanoparticles has great promise in drug delivery applications [23–25]. Moreover, it has been shown previously that by attaching a lipid moiety to a cationic CPP, the cellular delivery of siRNA can be dramatically improved [26, 27]. This enhanced activity is probably a result of increased complexation capacity in combination with increased endosomal escape. Based on these findings, INT may have contributed to improved transfection efficiency of mTat/PEI for gene delivery.

Cytotoxicity is one of the most important issues in biological application of the siRNA delivery system, and cytotoxicity may be associated with factors including stabilizer, structure, and zeta potential. It is known that cationic lipids and cationic polymers for gene delivery may cause toxic effect in vitro and in vivo [28–30]. Besides, the Tat is reportedly non-toxic both in vitro and in vivo [31], whereas cationic liposomes do show evidence of toxicity at high doses in vivo [32]. Cationic lipids are known to be membrane active [33]. They may therefore interfere with the membrane function and integrity of the cell or the subcellular compartments and lead to toxicity. However, the result of cell viability using HaCaT cells in this study indicated that INT/siRNA and mTat/PEI/INT/siRNA had little cytotoxicity. Compared to INT/siRNA, mTat/PEI/INT/siRNA showed significantly lower cytotoxicity at the highest concentration of siRNA used. Böttger et al. [15] reported that the transfection with INT demonstrated slightly higher though not significant level of toxicity in hepatocytes compared with L-iMAX. The most obvious difference between cationic lipids and cationic polymers is that the polymers do not contain a hydrophobic moiety and are completely soluble in water. Compared with cationic liposomes, they have the obvious advantage of compressing nucleotide molecules to a relatively small size [34, 35]. This can be crucial for gene transfer, as small particle size may be favorable for improving transfection efficiency. PEI, one of the most successful and widely studied gene delivery polymers, is a gene carrier with high transfection efficiency and a certain degree of cytotoxicity. Many factors affect the efficiency/cytotoxicity profile of PEI polyplexes such as molecular weight, degree of branching, ionic strength of the solution, zeta potential and particle size. To combine the advantages of both cationic polymer and liposome, water soluble lipopolymer was designed [22, 36]. This newly synthesized vehicle was a water soluble lipopolymer and non-toxic to a variety of cells. Based on the above data, the use of INT in combination with PEI presumably reduced cytotoxicity in the current study. Kafil et al. [29] showed that branched PEI can induce greater cytotoxicity than linear PEI in human epidermoid carcinoma cells. We used jetPEI, which is a linear PEI derivative in the present study, resulting in minimal cytotoxicity. Intriguingly, our results reveal that siRNA alone was most cytotoxic to HaCaT cells compared to the other complexes. In fact, siRNA itself may not be as safe as expected. High levels of siRNA have been known to result in the activation of innate immune responses and the production of cytokines in vitro and in vivo [37, 38]. Our results showed that complexation of siRNA with INT, and most notably, with mTat/PEI/INT can decrease toxic effect of siRNA itself. These findings indicate that the mTat/PEI/INT has a great potential not only for in vitro transfection reagent but also for clinical application as an in vivo siRNA delivery system with little cytotoxicity. In addition, mTat/PEI/INT/siRNA showed little cytotoxicity among several commercial reagents and X-tremeGENE and Lipofectamine 2000 shows significant higher cytotoxicity compared with the other reagents. Solanki et al. [39] reported Lipofectamine 2000 was extremely cytotoxic towards neural stem cells, which led to 95% cell death within 48 h of begin transfected with the negative control siRNA. Moreover, these transfection reagents are particularly known to active stress response pathways that might affect cell cycle regulation or metabolic signaling. Transfection reagent should be minimized transfection associated cytotoxicity otherwise these cytotoxicity leads incorrect interpretation.

After reaching the target cells, siRNA needs to interact strongly with the cell surface for a stable association, becomes internalized and reaches its cytoplasmic destination to integrate into RISC complexes. The native siRNA has little chance for a significant uptake on its own, and mainly relies on a carrier’s characteristics to overcome many obstacles. For example, cellular uptake of naked siRNA is extremely low due to its polyanionic nature [40]. A net positive charge of complex is necessary to ensure the uptake of complexes by cells carrying a negative charge. The present study revealed that zeta potential of mTat/PEI/INT/siRNA complexes was significantly higher than those of INT/siRNA and mTat/PEI/siRNA. It is notable that the zeta potential of mTat/PEI/siRNA dramatically increased after INT was mixed. The size of particles formed by carriers/siRNA is also an important characteristic for cellular internalization. The size of mTat/PEI/INT/siRNA complexes was significantly smaller than those of INT/siRNA and mTat/PEI/siRNA in this study. Interestingly, particle size was decreased after introduction of INT onto mTat/PEI/siRNA complexes. These factors including positive surface charge and small particle size must have contributed to the increased transfection efficiency. Our results were supported by other studies indicating that the amount of positive charges on the cell surface and the size of the vector/oligonucleotide complex played important roles in determining successful gene delivery [41–43]. A positively charged structure is desirable since it can preferentially adhere to the negatively charged cell surface receptors, leading to endocytosis. Non-viral vectors rely on the fundamentals of supramolecular chemistry in which anionic DNA and RNA molecules are condensed into compact, ordered nanoparticles that are ~ 50–200 nm in diameter, by complexing oligonucleotides with an appropriately designed cationic molecule [44–46]. The polycations reduce the size of the complex, and confer excess cationic charge to the complex, thereby enhancing their cellular uptake by an endocytosis pathway. Receptor-mediated endocytosis, pinocytosis, and phagocytosis are dependent on the size of the vehicle/gene complex [47–49]. Taken together, our results suggest that the charge and the particle size of the mTat/PEI/INT/siRNA complex may be the optimal conditions for internalization.

The cellular entry of siRNA may occur by direct transfer through cellular membranes or by energy-dependent membrane budding generally known as endocytosis. Endocytosis could occur through different pathways, including clathrin-mediated and clathrin-independent pathways, such as caveolae-mediated, clathrin- and caveolae-independent pathways and macropinocytosis, which are all involved in siRNA uptake depending on the nature of the carrier [50–52]. The internalization pathway could be different in different cells, and might even vary in the same cells depending on the experimental conditions. We used two specific inhibitors of endocytosis in the current study. Filipin III inhibits endocytosis by binding strongly to caveolae, present in the lipid raft domains of the cell membrane, thereby preventing lipid raft-mediated endocytosis [53]. β-Cyclodextrin have been used to deplete cells of plasma membrane cholesterol, leading to block in endocytosis of various toxins and glycosylphosphatidyl inositol-anchored proteins [54]. Chlorpromazine is an amphiphilic drug that prevents the recycling of clathrin proteins from endosomes back to the cell membrane thereby inhibiting the formation of new clathrin-coated pits [55]. Our results show that filipin III and β-cyclodextrin significantly inhibits the transfection efficiency and gene silencing of mTat/PEI/INT/siRNA in a dose-dependent manner with increasing concentration of filipin III and β-cyclodextrin, whereas chlorpromazine does not affect it. These results indicate that mTat/PEI/INT/siRNA might be internalized by caveolae-mediated endocytosis.

As almost all endocytic pathways are energy-dependent processes, they can be inhibited by low temperature incubation or ATP depletion [56, 57]. To analyze the energy-dependent endocytosis pathway, we used 4 °C for the treatment because cells consume less ATP and block the active transport at this temperature. The present study shows that the transfection efficiency with mTat/PEI/INT is significantly lower when transfection is carried out at 4 °C instead of 37 °C. The results indicate that low temperature directly inhibits transfection efficiency, suggesting that mTat/PEI combined with INT enters the cells in an energy-dependent pathway.

AFM has become a powerful tool for the visualization, probing and manipulation of RNA at the single molecule level [58]. AFM measurements can be carried out in buffer solution in a physiological medium, which is crucial to study the structure and function of biomolecules, also allowing studying them at work. In the current study, AFM analysis revealed the detailed morphology of siRNA and carrier/siRNA complex nanostructures. The mTat/PEI/INT/siRNA complexes appeared as almost spherical particles and the particle size was smaller than mTat/PEI/siRNA. These data were corroborated by DLS measurement. Further, an interesting finding was that the shape of the INT/siRNA was quite unique.

Using labeled siRNA to analyze the intracellular localization of siRNA in cells is a useful tool [59]. Visualizing siRNA localization in transfected cells and determining how siRNA localization correlates to RNAi activity is essential for understanding the mechanism of RNAi. Our results showed that complexation of mTat/PEI/INT with Cy5 labeled siRNA were more effective binding the cell membrane and leading to a distribution of the siRNA exclusively in the cytoplasm compared to other combinations. The result suggests that inhibition of gene expression by siRNA correlates with localization of fluorescently labeled siRNA in the cell. In consequence, this unique visualizing application can be used for tracking intracellular localization thus would have great potential for gene delivery and therapy.

## Conclusions

We demonstrated that Tat incorporated with histidine and cysteine residues and combined with cationic polymer and lipid results in much more efficient siRNA transfer and gene silencing with little cytotoxicity in vitro compared to several commercial transfection reagents. Our study also revealed that the internalization of mTat with PEI and INT is mediated by a caveolae-mediated and temperature-dependent endocytic pathway. Although the use of the combination of mTat/PEI with INT for gene delivery is considered efficient and relatively safe, long-term in vivo toxicity profiling is highly recommended as a next step. More importantly, the therapeutic potential of the delivery system using our novel hybrid vector should be assessed in a clinically relevant model. Although we used siRNA targeting *β*-*actin* to explore the effect of the novel delivery system, our findings open up the possibility of the hybrid vector using various siRNA targeting genes that cause a wide range of diseases.

## Methods

### Cell culture

HSC-3 human oral squamous cell carcinoma cells and HaCaT human keratinocytes were cultured in Dulbecco’s Modification of Eagle’s Medium (DMEM) supplemented with 10% fetal bovine serum (FBS; Life Technologies, Grand Island, NY), and 1% penicillin G (Life Technologies) with streptomycin sulfate. All cells were cultured at 37 °C in a humidified atmosphere with 5% CO_2_. This same medium was used for transfection.

### Non-viral vectors

To compare transfection efficiency of siRNA targeting *β*-*actin* (Santa Cruz Biotechnology, Inc. Dallas, TX) with several commercial reagents, Lipofectamine 2000 (Invitrogen, Carlsbad, CA), which is a cationic lipid-based transfection reagent, FuGENE HD (Promega Corp., Fitchburg, WI), which is a blend of lipids and other components, SuperFect (Qiagen, Valencia, CA), which is an activated dendrimer-based reagent, Lipofectamine RNAiMAX (Invitrogen), which is a cationic lipid-based transfection reagent, X-tremeGENE (Roche, Switzerland), which is a blend of lipids and other components, and INT (Polyplus-transfection, France) were used. Transfections of siRNA into cells were performed according to each manufacturer’s instructions. The HIV-1 Tat (RKKRRQRRRR) covalently fused with ten histidine and two cysteine residues (C-5H-Tat-5H-C), modified Tat (mTat) was obtained from Biomatik Corporation (Cambridge, Canada). To prepare mTat/siRNA complexes, the peptide solution (1 mM) and siRNA targeting *β*-*actin* was mixed in RNase-free water to several final volumes. The solution was quickly mixed for 5 s. The mixture was shaken vigorously for 60 min. The cap was opened intermittently to allow for air replenishment. The mTat/siRNA complex was added to PEI (Polyplus-transfection), and then incubated at room temperature for 60 min. Lastly, to prepare the mTat/PEI/INT/siRNA complex, INT in serum-free DMEM was mixed with the mTat/PEI/siRNA complex (described above) and incubated for 10 min at room temperature. Several volumes of the complexes were added into the plate.

### Quantitative real-time polymerase chain reaction with reverse transcription (QRT-PCR)

For *β*-*actin* mRNA expression, the total RNA in the harvested HSC-3 cells was isolated with RNeasy Mini Kit (Qiagen, Valencia, CA), according to the manufacturer’s instructions. The cells were homogenized in a lysis buffer. The lysis buffer containing the homogenate was centrifuged for 1 min at 13,000×*g* at 4 °C. The supernatant was applied to RNeasy column, rinsed, and eluted. RNAs were measured by NanoDrop ND-2000 Spectrophotometer (NanoDrop Technologies, Wilmington, DE) and cDNA was synthesized using a total of 1 µg RNA using QuantiTect^®^ Quantiscript reverse-transcriptase and RT Primer Mix (Qiagen), according to the manufacturer’s protocol. Real-time PCR was performed using Opticon Monitor 3^®^ (Bio-Rad, Hercules, CA) with Rotor-Gene™ SYBR^®^ Green (Qiagen), according to standard protocols. The sequences of *β*-*actin* and *glyceraldehyde*-*3*-*phosphate dehydrogenase* (*GAPDH*) specific primers are as follows: *β*-*actin* forward, 5′-GCCGAGGACTTTGATTGCAC-3′; *β*-*actin* reverse, 5′-ACCAAAAGCCTTCATACATCTCA-3′; *GAPDH* forward, 5′-ACACCCACTCCTCCACCTTT-3′; *GAPDH* reverse, 5′-TAGCCAAATTCGTTGTCATACC-3′. Standard curves were generated for each gene, and the amplification was found to be 90–100% efficient. The relative quantification of gene expression was determined by the comparison of threshold values. All the results were normalized to *Gapdh*. The results presented are the average of four replicate experiments. All the graphic data for mRNA expression are presented as the fold expression relative to the reference control cells.

### Enzyme-linked immunosorbent assay (ELISA)

The concentration of β-actin in cell lysate samples was evaluated by means of an enzyme-linked immunosorbent assay (ELISA). HSC-3 cells (10^5^ cells/well) were cultured into 24-well plates for overnight and treated by each transfection reagents for 48 h. Then, the cells were harvested using radio immunoprecipitation assay buffer (RIPA, Abcam, UK). Commercially available human ACTB/β-actin ELISA kit (LifeSpan BioSciences, Inc. Seattle, WA) was used to measure the levels of β-actin, according to the manufacturer’s instructions. The optical density was quantified using a multi-detection microplate reader, SpectraMax^®^ M5 (Molecular Devices, Sunnyvale, CA), at 450 nm wavelength.

### Cytotoxicity evaluation

Cytotoxicity of the transfection reagents was evaluated by 3-(4,5-dimethylthiazol-2-yl)-2,5-diphenyl tetrasodium bromide, MTT assay. HaCaT cells (10^6^ cells/ml) in 100 μl of DMEM supplemented with 10% FBS were seeded in 96-well plates and incubated overnight. Forty-eight hours after transfection with β-actin siRNA or vector/siRNA, the 5 mg/ml MTT reagent in 1× PBS (10 μl/well) was added into the plates and incubated for 4 h. After incubation, the medium was aspirated and dimethyl sulfoxide (50 μl/well) was added to stop the reaction. The optical density was quantified using a multi-detection microplate reader, SpectraMax^®^ M5 (Molecular Devices, Sunnyvale, CA), at 540 nm wavelength. The percentage of cell viability was calculated by comparing the appropriate optical density to the control cells, which were not transfected.

### Zeta potential

Zeta potentials of each complexes were measured at 25 °C by a Zetasizer Nano ZS90 (Malvern Instruments Ltd, UK). This instrument is equipped with a red laser of wavelength 630 nm and measures the electrophoretic mobility of the particles using phase analysis of scattered light in an experimental set up similar to Laser Doppler Velocimetry (M3PALS technique, Malvern Instruments Ltd). Zeta potential was derived from the electrophoretic mobility using the Smoluchowski model since the measurements were performed in aqueous solutions of moderate ionic strength (i.e. electrical double layer thickness ≪ the particle size). Each sample was observed with 20 repeated measurements across 3 trials.

### Dynamic light scattering

The size distribution of β-actin siRNA and the vector complex was measured by dynamic light scattering (DLS), using a Zetasizer Nano ZS90 equipped with a red laser of wavelength 630 nm. Scattered light intensity was measured at 90° and particle size was calculated from autocorrelation data analysis by Zetasizer Nano software. All solutions used were filtered with 0.22-µm filters immediately preceding sample preparation. Each sample was observed with 20 repeated measurements across 3 trials.

### Effect of temperature on transfection

To determine if the functional delivery of siRNA complex is an energy-dependent process, experiments were performed at two temperatures (4 °C and 37 °C). HSC-3 cells were incubated at 4 °C and 37 °C for 20 min and then transfected with/without siRNA or siRNA complex at the same temperatures for 3 h. The cells were washed with PBS, replenished with fresh media, and incubated for 48 h at 37 °C prior to RNA isolation. The gene expression was analyzed by QRT-PCR. Data was calculated as % changed transfection efficiency compared to the control cells incubated with siRNA without vectors.

### Treatment with endocytosis inhibitors

HSC-3 cells were treated with filipin III (1, 2, and 3 μg/ml), β-cyclodextrin (1 and 5 mM), or chlorpromazine (5 and 10 μg/ml) (all of them from Sigma-Aldrich) in DMEM medium with 10% FBS for 30 min at 37 °C. Subsequently, siRNA targeting *β*-*actin* and the vector complex was added and incubation was continued for 3 h. Then, the cells were washed with PBS and regular medium with serum was added. After 48 h incubation, gene expression was analyzed by QRT-PCR. Data was calculated as % changed transfection efficiency compared to the control cells incubated with siRNA without vectors and the inhibitors, filipin III, β-cyclodextrin, or chlorpromazine.

### Atomic force microscopy

The nanostructure of vector/siRNA complexes was imaged on atomic force microscopy (AFM). Briefly, AFM images were obtained on MFP-3D AFM microscope (Oxford Instruments Asylum Research, Inc., Santa Barbara, CA) under acoustic AC mode using Si probes operating at a resonant frequency of 154 kHz. All measurements were carried out at room temperature and acquired images had a resolution of 512 × 512 pixels collected at a speed of one line min^−1^. Freshly cleaved mica surface was used as the substrate for imaging. To acquire images, about 50 µl of the prepared sample was pipetted on to the mice surface and allowed to interact with the surface for 5 min. Mica was then washed with RNase-free water to remove unattached complexes. After air-drying, the mica surface was analyzed by MFP-3D AFM at room temperature using the tapping mode. Post image processing of AFM images was done using the IGOR Pro 6.37 software (WaveMetrics, Lake Oswego, OR).

### Confocal microscopy

Fluorescent labeling and staining were performed to evaluate cellular distribution of labelled siRNAs and the particles. For confocal microscopy, HSC-3 cells were seeded onto glass coverslips in 24-well plates and incubated for overnight at 37 °C in a humidified atmosphere with 5% CO_2_. *β*-*actin* siRNA fluorescently labelled with Cy5 dye using the Label IT^®^ siRNA Tracker Intracellular Localization Kits (Mirus Bio LLC, Madison, WI) and were added to cells with each transfection regents. All transfection was performed according to the previous protocol and incubated for 24 h. The cells were then fixed with 4% paraformaldehyde and washed with Hank’s balanced salt solution (HBSS), and then stained for 10 min at 37 °C with CF™ 488A (Alexa Fluor 488) Wheat Germ Agglutinin (5 µg/ml, Biotium, Inc. Fremont, CA) and DAPI (300 nM, Thermo Fisher Scientific, Waltham, MA). The cells were washed with PBS and sealed in ProLong™ Gold antifade reagent (Invitrogen) before visualizing with a 63×/1.4 NA oil immersion objective under a Carl Zeiss LSM880 laser scanning microscope system (Carl Zeiss, Oberkochen, Germany).

### Statistical analysis

Descriptive statistics were computed for each experimental condition to summarize the mean expression levels. Bivariate comparisons (ANOVA and t-test) were computed to compare different experimental conditions. In instances where multiple comparisons testing were required, the Tukey test for multiple comparisons was utilized. For all statistical analyses, *p* values less than 0.05 were considered significant. Results were presented as mean ± standard error of the mean (SEM).

## Abbreviations

RNAi: RNA interference
siRNA: small interfering RNA
RISC: RNA-induced silencing complex
CPP: cell-penetrating peptides
HIV-1: human immunodeficiency virus type-1
mTat: modified Tat
PEI: polyethylenimine
INT: INTERFERin
DMEM: Dulbecco’s Modification of Eagle’s Medium
QRT-PCR: quantitative real-time polymerase chain reaction with reverse transcription
GAPDH: glyceraldehyde-3-phosphate dehydrogenase
DLS: dynamic light scattering
AFM: atomic force microscopy
SEM: standard error of the mean
